# «One prick and then it´s done»: a mixed-methods exploratory study on intramuscular injection in heroin-assisted treatment

**DOI:** 10.1186/s12954-021-00584-3

**Published:** 2021-12-18

**Authors:** Maximilian Meyer, Ramón Eichenberger, Johannes Strasser, Kenneth M. Dürsteler, Marc Vogel

**Affiliations:** 1 Division of Substance Use Disorders, University of Basel Psychiatric Clinics, Wilhelm Klein-Strasse 27, 4002 Basel, Switzerland; 2 Faculty of Medicine, University of Basel, Klingelbergstrasse 61, 4056 Basel, Switzerland; 3 Department for Psychiatry, Psychotherapy and Psychosomatic, University of Zurich, Lenggstrasse 31, 8008 Zurich, Switzerland; 4 Psychiatric Services of Thurgovia, Division of Substance Use Disorders, Seeblickstrasse 3, 8596 Münsterlingen, Switzerland

**Keywords:** Intramuscular substance use, Harm reduction, Injecting substance use, Opioid agonist treatment, Opioid injection

## Abstract

**Background:**

Intramuscular (IM) injection of drugs is associated with high rates of injecting-related injuries and diseases. However, little is known about the role of this route of administration in heroin-assisted treatment. The aim of this study was to determine the prevalence of IM diacetylmorphine administration and associated complications as well as to explore patients’ views and opinions on the topic and the underlying reasons for this practice.

**Methods:**

The research site was a Swiss outpatient treatment centre specialised in heroin-assisted treatment. We conducted in-depth interviews with two patients who intramuscularly inject diacetylmorphine. Interviews were analysed qualitatively, and emerging themes were used to develop a 38-item questionnaire on IM injections. We then offered this questionnaire to all patients in the treatment centre.

**Results:**

Five main themes emerged from the in-depth interviews: poor venous access, side effects, subjective effects, procedure for IM injection, and consideration of alternatives to IM. These themes covered the rationale for using this route of administration, complications, subjective effects of IM diacetylmorphine, hygiene and safety measures as well as alternative routes of administration. Fifty-three patients filled in the questionnaire. The lifetime prevalence of IM injections was 60.4% (*n* = 32) and 34.4% (*n* = 11) of the patients stated that IM injection was their primary route of administration. No participant reported using the IM route for street drugs. The main reason for IM injections was poor vein access. Other reasons given were time saving and less risk of injuries. Complications included induration of muscle tissue and pain, whereas more severe complications like thrombosis and infections of the injection site were reported much less often.

**Conclusion:**

As the population of opioid-dependent individuals is aging and the deterioration of access veins is likely to increase, the frequency of IM injecting will equally increase. Even though our data show that the IM injection of diacetylmorphine in a clinical setting is a common practice and appears to be relatively safe, research on alternative routes of administration is needed to provide potentially less harmful alternative routes of administration in heroin-assisted treatment.

## Background

Heroin-assisted treatment (HAT) has been introduced in Switzerland in 1994 for patients with very severe opioid use disorder [1]. It comprises the use of pharmaceutical heroin (diacetylmorphine, DAM) in patients for which traditional opioid agonist treatment (OAT) like methadone maintenance treatment was previously not successful. DAM is approved for oral and intravenous (IV) administration. Patients who seek the euphoric effects (“high”) of rapid-onset opioids prefer IV injection. However, this route of administration is becoming increasingly unsuitable for a large part of the opioid-dependent population in Switzerland, as long-term IV opioid use is associated with deterioration of access veins [2, 3]. Therefore, patients in HAT who are not able to inject IV anymore but do not like to relinquish the euphoric effects associated with the rapid onset of DAM often resort to off-label intramuscular (IM) injection [4].

Pharmacokinetics differ substantially depending on the route of administration. Peak plasma concentrations of IM DAM are significantly lower than those reached by IV DAM [5]. However, due to the sustained release in the circulatory system following IM injection, there are prolonged and high levels of DAM and its metabolites, resulting in a three- to fourfold higher area under the plasma concentration–time curve. Hence, IM DAM might be well-suited to reduce craving and withdrawal symptoms [5].

Literature on IM injection in the context of substance use is surprisingly scarce. It has been suggested that IM injection for recreational purposes is a rare phenomenon and rather perceived as “missed hit” [6]. A broad range of injecting-related injuries and diseases (IRID) like bacterial skin infections, chronic wounds and ulcers are associated with IM injection [7–9]. Other IRID include potentially life-threatening conditions like endocarditis, sepsis, bone and joint infections as well as thrombosis and emboli [10]. Compared to IV injection, higher incidences of abscesses and cellulitis have been observed [11, 12].

IM injecting is also practised by patients in HAT, in whom it is prescribed off-label [4]. However, the prevalence of this practice is unclear, and no literature exists on the complications associated with medically supervised IM injections of DAM. It has also never been investigated why HAT patients choose this route of administration and whether there are reasons for it beyond the poor vein access. As IRID are responsible for a substantial part of the health-care cost generated by the treatment of physical illnesses in individuals who inject substances [13], a clearer understanding of IM injections could help to prevent these complications.

To our knowledge, there is no study on IM injection in HAT available and patients’ views and experiences have not been explored. Moreover, no data exist on the prevalence, frequency, and complications of IM injections in HAT. Furthermore, the injecting procedure has not yet been described. This is surprising, as off-label prescriptions of IM DAM in HAT are no longer isolated cases. Understanding the mechanisms and underlying factors of IM injections is paramount to improve safety measures, to explore a possible need of alternative routes of administration in accordance with the principles of harm reduction, and to inform guidelines on treatment with injectable opioids. Therefore, this study aimed to explore patients’ views on and experiences with IM injections in HAT.

## Methods

### Setting, design, and procedure

The study was conducted in an outpatient treatment centre of the Psychiatric University Clinics in Basel, Switzerland, specialised in HAT. We used a mixed-method exploratory design combining qualitative and quantitative approaches [14]. In a first step, two in-depth interviews were conducted with patients who regularly injected DAM IM in order to explore subjective experiences, complications, and ideas concerning IM injections. Table 1 provides the most relevant questions and topics addressed in the interviews. RE conducted interviews in Swiss German and transcribed them into standard German as no written standard of Swiss German exists. RE, KMD, and MV conducted thematic analysis of the interviews. In a second step, the emerging themes of the interviews were used to develop a 38-item questionnaire. This questionnaire was piloted with one of the interviewed patients and adapted accordingly to improve comprehensibility and clarity and to reduce ambiguity. In the final step, we recruited a convenience sample of patients in HAT. Inclusion criteria were participation in HAT, aged between 18 and 65 years, and willingness to participate. Patients with insufficient knowledge of the German language or severe neurocognitive impairments that could affect completion of the questionnaire were excluded. All other patients of the centre were offered participation.

**Table 1:** Interview topic guide

1	Which routes of administration do you use for DAM and illicit substances?
2	What is the reason for the respective route of administration and in particular the reason for the intramuscular (IM) injection? Did the reasons change over time?
3	When did you start injecting IM? How did you find out about it? Is IM injecting your primary route of administration? Was it necessary to adjust the dose?
4	How do you rate IM injection in general and when compared to other routes of administration?
5	Provide a description of the IM injection procedure. Where are you injecting? What do you look out for during IM injections?
6	Please describe the effect of IM DAM compared to other routes of administration you have used
7	Did you ever experience any complications in connection with IM injections? Which ones?
(8)	In case of no experiences with IM injections: Why did you refrain from ever injecting intramuscularly?

Selected quotes were translated into English by MM and translated back to German by MV to ensure that the content remained consistent.

Descriptive statistical analysis was performed with SPSS version 23.0 for Macintosh.

## Results

### Interviews

One male (P1, 59 years old, two daily IM injections) and one female patients (P2, 48 years old, one daily IM injection) were interviewed about the IM injection of DAM. Multiple themes emerged during the interviews, highlighting the reasons, complications, and subjective effects in connection with this route of administration.

#### Theme 1: poor venous access

Both patients stated that the condition of their access veins had deteriorated due to prolonged use of intravenously injected street heroin, methadone, and DAM. It emerged that IM injection was not their route of choice but rather served as an alternative to IV injections. The latter became inconvenient as it took too much time, was accompanied by multiple attempts and thus injuries, and often did not yield success in finding a vein and getting the “rush”. Interestingly, Patient 2 stated that she would not want to return to regular IV injections, whereas Patient 1 wished to return to IV injections if the condition of his veins ever allowed it.

“At first I still tried to [inject] intravenously which worked occasionally. But even so, I had such bad veins, that I switched then. At first subcutaneously, but then it was already too much for subcutaneous [injection], then I switched to intramuscular. […] I have no other choice. […] If I could, I would do it intravenously. […] The reason [for IM injection] was clearly that I didn’t have veins anymore.” (P1)

“And that you just don´t fancy maltreating your body for an hour with the [needle] prick. And in the muscle, you make one prick and then it’s done.” (P1)

“…I received 30 [mg DAM] and did not find any veins. And then here a droplet, there a droplet and in the end, I had a lot of holes in the body but didn’t get anything out of it. That was when I started to intentionally inject in the muscle, because – well, it is also inside the body. […] It’s not as if, just because we are injecting, that we think it’s so great to inject ourselves. […] If I had still have had veins, I wouldn’t have switched [to IM injection].” (P2)

“That in between you think ‘a rush like that would be nice right now’. That’s why I say, if I had the choice, I could imagine that I would tend to stay with the intramuscular (injection), but especially on a weekend, on Saturday evening or on weekend mornings […] I [inject DAM intravenously] and have this rush and can enjoy it for ten minutes.” (P2)

#### Theme 2: side effects

When asked about complications and drawbacks of IM injections, patients reported indurations of muscle tissue, pain, skin rashes, the accidental hitting of a nerve, and accidental IV injections. Regarding the latter, preferences differed between the interviewed patients. Patient 2 stated accidental IV injections to feel unpleasant. It also emerged that patients were aware of the elevated risk of infection when injecting street heroin as it is more likely to be contaminated. However, despite the long time period for which both patients had injected IM during their participation in HAT (i.e. 21 years patient 1 and 10 years patient 2), neither had suffered from related abscesses or major infections and reported experiencing only minor problems. Additionally, both patients reported that they had never intentionally injected street heroin intramuscularly.

“And I can only do that [IM injection] now because I do it here at Janus [i.e. the treatment centre]. I couldn’t do it on the street. With the dirt [illicit heroin] that is on the street, you would immediately get an inflammation, you just can't inject it in the muscle. Over time there’s also hardening and stuff, you have to massage it out […].” (P1)

“Yes, this (pain) occurs sometimes. Most of the time, you have a little bit of an allergic reaction, that happens sometimes. Then you get […] on the skin- you get little pustules.” (P1)

“That happened to me before, (I was) accidentally hitting a nerve. That is like an electric shock. […] That can happen sometimes. I have experienced this two or three times up until now.” (P1)

“I have already heard that abscesses can occur. But I don’t know what- they can occur in general.” (P2)

“It happens sometimes that I accidentally hit a vein. That's okay, too. Then I don't pull it out because ‘shit, now I have a vein’. […] it´s sometimes unpleasant when I hit a vein unintentionally because then I [inject], and it shoots into my brain, and I don't expect it. Yes, then it itches depending on that. There is a sort of needling.” (P2)

#### Theme 3: subjective effects

When asked about the subjective effects of IM DAM, patients described a significant difference to IV DAM. Most notably, the delayed onset of drug action, the prolonged duration, and a milder “rush “, were mentioned.

“It is not exactly the same. […] Intramuscular, in the muscle, it takes a couple of minutes until you start to notice it. So, mostly when I [inject] here, I notice it fully when I am at home. It is not as instantaneous, as [injecting] in the veins. For example, in the veins you notice it [the effect] immediately after a few seconds.” (P1)

“I have the feeling intramuscular [DAM] lasts even longer. When you inject into the veins, it’s like ‘Whack, boom’, the flash, so to speak […]. You don’t have that when you inject intramuscularly. The [effect] doesn’t come. But rather it comes slowly and yes, you feel warm, I don’t know, it's difficult to describe. Warm, comfortable, a pleasant feeling somehow. And that just comes slowly. And I feel it lasts for a while [longer].” (P2)

#### Theme 4: procedure for IM injection

Patients described their use of hygiene measures (disinfection), safety measures (needle aspiration), and the regular switching of injection sites. Both noted the importance of choosing the correct needle size and length. Patient 2 noted that female patients in general feel less comfortable taking off their clothes and are therefore more likely to be limited in the rotation of injection sites.

“If I were to explain it [IM injections] to someone, I would say that they should make sure that the needle is long enough. Many people use needles that are too small, I think. […] I inject it [DAM] relatively slowly. I just do it that way, I don't know why.” (Patient 1)

“Sometimes indurations occur, especially on the upper arms because I don't feel like pulling down my trousers in here […] they [clinic staff] always said [inject] in the upper leg or in the upper arm but I think the doctor tells people in the upper leg. But especially with women there is this problem. They are more embarrassed about having to take off their trousers and I also find it gross to sit on these chairs with my bare legs.” (Patient 2)

#### Theme 5: consideration of alternatives to IM

When asked about alternative routes of administration, patients noted that they listed injecting in the inguinal vein and nasal administration (sniffing). Patient 2 stated that she might try nasal DAM in the future, whereas both patients were opposed to inguinal injections.

“I never did that, the groin. That's not an option. Neither is nasal. Well, it was in the past, but here in the programme it's only IV, IM or oral. […] I've never [injected] into the groin and I've never [sniffed] it.” (Patient 1)

“One client here sprays it into her nose like with one of those atomisers. That's the first time I've seen that before. […] The groin is not without danger, I mean, to inject into such a big vein and if you then catch an inflammation there- absolutely not for me, no. […] [Nasal DAM] would be worth a try, right?” (Patient 2)

### Questionnaire

At the time of study conduction, 155 patients received HAT at the research site, and all were offered participation. Fifty-three patients decided to participate and fill out the questionnaire. There was no compensation for participating. The sample characteristics are presented in Table 2 and are representative of Swiss patients in HAT [15].

**Table 2:** Sample characteristics (*n* = 53)

	*M*	SD
Age in years	47.51	8.38
Age at time of first opioid use (*n* = 51)	19.2	5.3

	*N*	%
Sex		
Female	19	35.8
Male	34	64.2
Family history of mental disorders (*n* = 52)	29	55.8
Alcohol use disorder	22	42.3
Other substance use disorder	16	30.8
Other psychiatric disorders	11	21.2
Currently employed	25	47.2
Duration of HAT in years		
< 1	3	5.7
1–5	10	18.9
6–10	11	20.8
11–15	10	18.9
16–20	6	11.3
> 20	13	24.5

*SD* standard deviation, *M* mean; sample sizes differ due to missing data

#### Reasons for, prevalence, and procedures of intramuscular injections

More than half of our sample (60.4%, *n* = 32) reported IM injecting at least once in their lifetime. Of those, 84.4% (*n* = 27) stated to have done it intentionally and 34.4% (*n* = 11; 20.8% of the total sample) chose IM injections as their primary route of DAM administration. Only three respondents (9.4%) had injected IM outside of the treatment setting. Table 3 displays a comprehensive overview of responses on injection habits.

**Table 3:** Prevalence and habits of IM injection

	*n*	%
Lifetime prevalence of IM injection (*n* = 53)	32	60.4
Female (*n* = 19)	13	68.4
Male (*n* = 34)	19	55.9
How did you learn about IM injection? (*n* = 32)		
Staff	23	71.9
Friends/other patients	9	28.1
Found out by myself	4	12.5
Education	1	3.1
Unknown	1	3.1
Length of IM injection experience (*n* = 30)		
< 1 year	17	56.7
1–5 years	6	20.0
6–10 years	6	20.0
11–15 years	1	3.3
Current frequency of IM injecting (*n* = 32)		
Several times a day	5	15.6
Once daily	3	9.4
Several times a week (2–6x)	3	9.4
Several times a month (2–3x)	1	3.1
Rarely	5	15.6
No longer at all	15	46.9
Injection sites (*n* = 31)		
Upper leg	19	61.3
Upper arm	18	58.1
Lower leg	3	19.4
Feet/hand	2	6.5
Abdominal region	1	3.2
Forearm	1	3.2
Regular rotation of injection site (*n* = 31)	24	77.4
Frequency of rotation (*n* = 23)		
Daily	16	72.7
Weekly	4	18.2
Rarely	3	13.6
*Reasons for IM injection given by participants who had previously injected intramuscularly (*n* = 32)		
Bad condition of access veins/impossibility of intravenous injection	25	78.1
Time saving (no need to locate veins)	13	40.6
Less injuries (one prick instead of multiple intravenous attempts)	10	31.3
Avoiding intravenous injection	3	9.4
Longer-lasting effect compared to intravenous injection	3	9.4
Less complications	2	6.3
Higher level of functioning after injection	2	6.3
Trying it out	1	3.1
Physical illness	1	3.1
Delayed onset	1	3.1
Doing it out of habit	0	-
Reduced risk of overdose	0	-

Sample sizes differ due to missing data

*Multiple answers were possible

All participants who chose IM injections as their primary route of administration had participated in HAT for more than 6 years and were 31 years of age or older (M = 48.36, SD = 7.32). The main reason given for use of IM injections was “bad condition of access veins/impossibility of intravenous injection” (78.1%, *n* = 25). Other reasons included “time saving”, “less injuries”, and the longer-lasting effect of DAM. No respondent chose “reduced risk of overdose” (see Table 3). Three patients reported IM injection despite being principally able to inject IV.

Fifteen respondents gave reasons on why they stopped IM injections. The most common was “insufficient effect” (*n* = 6), followed by “availability of access veins” (*n* = 4), “other” (*n* = 3), and “pain/induration” (*n* = 2). When asked about whether the DAM dose needed to be adjusted after changing from IV to IM injection, 72.2% of 11 respondents (*n* = 8) answered that adjustment had been necessary. Seventy-five percent (*n* = 6) reported that they needed a lower dose, but 25% (*n* = 2) reported that their dose had to be increased.

When asked about accidental IV injections when intending to inject DAM in the muscle, 82.8% of 29 respondents (*n* = 24) stated having experienced this and 69.2% of 26 respondents (*n* = 18) stated it to be a pleasant experience. When asked, whether these accidental IV injections were tolerated (i.e. if intoxication occurred or it was felt that the subjective effect was stronger than preferred), 68.8% of 32 respondents (*n* = 22) answered yes, 12.5% (*n* = 4) answered no, and 18.8% (*n* = 6) of the respondents answered that they did not know.

Regarding overdose events, 41.9% (*n* = 13) of 31 respondents answered to have had experienced such an event, but only 16.7% (*n* = 2) stated that it happened due to IM injection.

#### Subjective effects of intramuscular DAM injections

**Fig. 1:**
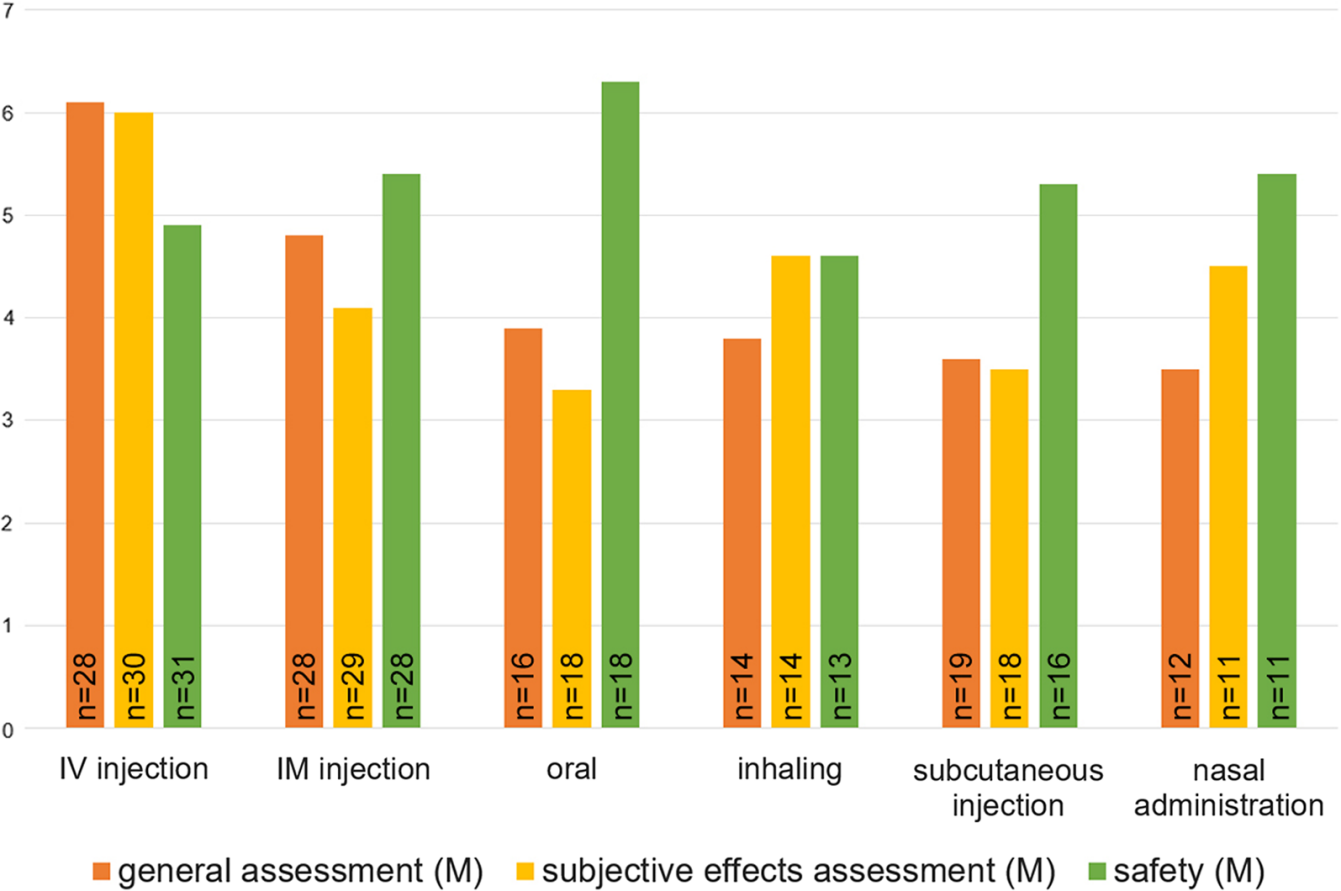
Assessment of routes of administration and the effect of DAM for each route. Legend: *M* = mean; routes of administration were rated on a 7-point Likert scale. 1 = very bad/no effect; 7 = very good/very intense effect. Safety was rated between 1 = very frequent occurrence of long-term harm and 7 = very rare occurrence of long-term harm. Participants only rated routes that they had previously employed; therefore, n ranged from 30 to 11

We asked patients’ estimates of onset and duration of action following IM injections. About half of the respondents (52%, *n* = 13) estimated the effect of DAM to last longer than after IV administration. We also asked about which route of administration produced the most similar effects to IM injection. Responses are provided in Table 4. Participants also gave a general rating, a rating of the subjective effects of each route well as a subjective assessment of long-term harm on a 7-point Likert scale (Fig. 1).

**Table 4:** Subjective effects of IM injected DAM

	*n*	%
Estimation of onset of action (*n* = 31)		
0–3 min	3	9.7
5–10 min	16	51.6
10–15 min	6	19.4
> 20 min	5	16.1
°Estimation of duration of action (*n* = 28)		
0.5–6 h	5	17.9
8–12 h	19	67.9
12–18 h	2	7.1
20–24 h	2	7.1
Route of administration that resembles IM injection the most (*n* = 31)		
IV	11	35.5
Subcutaneous injection	6	19.4
Inhaling	4	12.9
Nasal administration	4	12.9
None	4	12.9
Oral	2	6.5

°Summary of free text inputs

#### Complications and risk of long-term harm related to intramuscular injection

Complications had been experienced by 43.8% (*n* = 14) of IM injecting respondents. Induration of muscle tissue was the most common (71.4%, *n* = 10), followed by pain at injection site (36%, *n* = 5) or in the affected muscle (36%, *n* = 5). Thrombosis and accidental intra-arterial injection were not reported. As reported previously, two participants stated to have experienced an overdose event in connection with IM injection. Table 5 provides an overview of reported complications and preventive measures applied by participants to avoid the former.

**Table 5:** Experienced complications

	*n*	%
*Experience with complications (*n* = 32)	14	43.8
Indurations (*n* = 14)	10	71.4
Pain at injection site (*n* = 14)	5	35.7
Muscle pain (*n* = 14)	5	35.7
Haemorrhage (*n* = 14)	3	21.4
Infection (*n* = 14)	1	7.1
Hitting nerves (*n* = 14)	1	7.1
Intra-arterial injection (*n* = 14)	–	0
Thrombosis (*n* = 14)	–	0
*Preventive measures (*n* = 31)		
Disinfection	25	80.6
Rotation of injection sites	19	61.3
Massaging	11	35.5
Needle aspiration	6	19.4
Skin care products	1	3.2

*Multiple answers were possible

Long-term harm of IM injection was estimated to be less frequent when compared to subcutaneous, IV, and inhaled administration. Oral administration was estimated to be the safest (Fig. 1). However, we did not calculate inferential statistics.

Respondents who had never injected intramuscularly (*n* = 19) were asked for the reasons for not doing so. Seventy-four percent (*n* = 14) stated that they preferred a different route of administration (IV: 42.9%, *n* = 6; nasal and oral: 21.4%, *n* = 3; inhaling: 7.1%, *n* = 1). Other answers given were “insufficient effect” (*n* = 6), “fear of complications” (*n* = 4), and “pain” (*n* = 2).

## Discussion

To our knowledge, this is the first study investigating prevalence and subjective patient experiences of the IM injection of substances. Our results illustrate that IM injection is in fact a common route of administration in HAT patients, as more than half of our sample had experience with IM injections. The paucity of existing research on this topic is in stark contrast to its obvious relevance in injectable opioid agonist treatment.

Five themes emerged in our in-depth interviews, providing the basis for the questionnaire items. The in-depth interviews suggested that the attitude towards IM injections differs greatly among patients. Though both interviewed patients stated they had started IM injections due to deterioration of their access veins, one patient stated that he would return to IV injections if he could, while the other patient stated that she likely would not return as she got used to the milder effects of IM DAM. We found the bad condition of access veins to be the main reason for IM injection of DAM in our sample. This finding was expected as the trauma of repeated injections can lead to scarring, sclerosis, and thrombosis of veins [16]. Heroin acidity itself is also discussed as potential etiological mechanism of venous sclerosis, putting individuals with chronic injecting substance use at high risk of venous deterioration [3]. Additionally, the patient population in OAT and HAT in Switzerland is aging [17, 18]. It is likely that with increasing age the number of patients with a deteriorated vein status is equally growing, contributing to the important role of IM injection in HAT. Interestingly, participants who had experience with IM injections seem to judge them to be associated with less long-term harm as compared to IV administration. This might be the case because most patients reported the previous deterioration of the venous system before initiating IM injection. However, the general preference for IV over IM injection remained. Most patients reported accidentally hitting a vein as a pleasurable experience, while one of the qualitative interviewees described the unexpected rush as disturbing. This illustrates the individual differences concerning the routes of administration, which need to be considered in clinical practice. Our findings also underline the importance of measures to improve and sustain the status of peripheral veins during treatment with injectable opioid agonists. Studies on the effectivity of injectable OAT (iOAT) have so far not taken the route of administration into account. Given the significant role this practice seems to play, future studies should also consider IM injections. They should assess patients’ preferences for routes of administration and the role that these play for treatment success, i.e. is IM injecting as effective as IV? Does treatment simply continue when patients switch from IV to IM or is this a point in time where patients drop out of treatment?

In our interview, Patient 2 stated that female patients might generally feel less comfortable disrobing at the treatment centre. This is relevant, as it limits female patients in the rotation of injection sites, putting them at a higher risk of venous deterioration or side effects of IM injections. This issue should be considered by health-care decision makers planning to implement iOAT and iOAT providers. It could be addressed by offering separate time slots for dispensing or by providing a separate dispensing room for affected patients.

Although many participants had practiced IM injecting for years, less than half reported IM-injection-related complications. Indurations, pain, and haemorrhages appear to be most common, whereas more severe complications like infection or thrombosis were reported much less often. This is in contrast to the findings in the literature on IM use of street drugs, which report the lifetime prevalence of bacterial infections like cellulitis and abscesses to be much higher [11]. Patients also reported application of a range of methods to prevent complications, among them hygiene, rotation of injection sites, and massage of indurations. We conclude that injections under medical supervision and high levels of hygiene in outpatient HAT centres seem to prevent many complications, therefore improving the safety of off-label IM DAM use. In line with this, only three of our patients reported to have injected intramuscularly outside of the treatment setting in their lifetime, suggesting a larger role for IM injection in therapeutic vs. non-therapeutic settings. IM injections in the latter setting are likely to be “missed hits” [6]. Likewise, reports of overdose after IM injection were uncommon. The lower maximum blood levels for DAM after IM compared to IV injections described in the pharmacological literature [5] could well explain this finding along with the supervised setting. Interestingly, we found that an adjustment of the injected DAM dose was necessary in most patients following the switch from IV to IM. Whereas the majority reported to have lowered their dose, some patients increased it. This could be explained by the sustained release of DAM following IM injection, resulting in a larger area under the plasma concentration–time curve when compared to IV [5]. Patients who seek the “high” of injected DAM might therefore increase their dose, whereas patients less intent on the “high” or primarily seeking to prevent withdrawal would not adjust or lower their dose.

There is a clear need for prospective studies that compare the frequency of adverse events of different routes of administration in HAT. Furthermore, very few or no data exist on the prevalence of IM injection in other settings (e.g. safe injection rooms) or populations in other countries. Our data also underline the need for the evaluation of alternative, less harmful novel routes of administration. Only a third of patients with IM injection experience used IM injections as their main route of administration, most of whom had previously injected intravenously. IM injections are therefore not their first choice, and alternative, less invasive routes of administration may improve treatment convenience and prevent adverse effects. Early experimental and pharmacological data suggest similar effects of nasal DAM administration when compared with IM injection [19]. However, in our study, participants rated the subjective effects of IM DAM injection to be more similar to IV injection than nasal administration. It may have played a role for these differences that participants provided the assessments of the different routes at a time point independent from when they last administrated DAM/heroin. In an experimental setting, these ratings are given in close temporal proximity. Nevertheless, recent case reports demonstrate that nasal DAM may in fact be viable and safe [20], and future studies should assess whether IM injecting patients could benefit in particular from this treatment approach.

Our findings may be useful to develop recommendations on the circumstances, requirements, and procedures surrounding IM injections in HAT. Hopefully, further research will contribute to the evidence-based refinement of such recommendations in order to improve treatment of this marginalised patient population.

### Strength and limitations

We did not conduct inferential statistics with the quantitative data due to the small numbers and the exploratory nature of the study. Furthermore, due to the retrospective study design, answers could be subject to recall bias. Also, the generalisability of our findings might be impaired as patients with IM injection experience may have been more likely to participate than others, resulting in a potential overrepresentation of IM injecting patients. The reported prevalence of IM injecting patients should therefore be interpreted cautiously.

The combination of interviews informing the design and subsequent use of a questionnaire is a strength of our study as it allows the investigation of this under-researched topic and the generation of further research questions.

## Conclusions

Surprisingly few studies exist on IM substance use in general, and we found no studies reporting on IM injection in injectable OAT. However, our data show that, while often not the preferred option, IM injections are a common practice in HAT. As the population of opioid-dependent individuals is aging, the prevalence of venous deterioration will likely further increase and research on alternative routes of administration will become even more relevant. We found that IM injecting is in principle safe due to medical supervision, hygiene standards, and patient education. Further, prospective research with larger sample sizes is needed for the development of evidence-based guidelines. In addition, further studies evaluating the effects and feasibility of nasal DAM as compared to IM DAM are required to provide more suitable, safe, and convenient treatment approaches for the aging population of patients in HAT. However, our data may serve as a basis for the creation of pragmatic recommendations regarding the handling and procedures of IM injections in HAT.

## Data Availability

The dataset supporting the conclusions of this article is available from the corresponding author upon reasonable request.

## Abbreviations

HAT: Heroin-assisted treatment
DAM: Diacetylmorphine (heroin)
OAT: Opioid agonist treatment
iOAT: Injectable opioid agonist treatment
IV: Intravenous
IM: Intramuscular
IRID: Injecting-related injuries and diseases
